# Impact of Electronic Nicotine Delivery Systems on Oral Mucosa: A Cytopathological and Molecular Study

**DOI:** 10.1111/jop.70095

**Published:** 2025-11-27

**Authors:** Alba Pérez‐Jardón, Bruna Fernandes do Carmo Carvalho, Cintia Micaela Chamorro‐Petronacci, Maria Dolores Reboiras‐López, Natalia de Carvalho Faria, Renata Falchete do Prado, Mónica Ghislaine Oliveira Alves, Fábio França Vieira e Silva, Maria‐Elena Padín‐Iruegas, Mario Pérez‐Sayáns, Janete Dias Almeida

**Affiliations:** ^1^ Oral Medicine, Oral Surgery and Implantology Unit (MedOralRes), Faculty of Medicine and Dentistry University of Santiago de Compostela Santiago de Compostela Spain; ^2^ ORALRES Group, Health Research Institute of Santiago de Compostela (FIDIS) Santiago de Compostela Spain; ^3^ Universidade Estadual Paulista (UNESP), Instituto de Ciência e Tecnologia São Paulo Brazil; ^4^ Instituto de los Materiales de Santiago de Compostela (iMATUS) Santiago de Compostela Spain

**Keywords:** biomarkers, electronic nicotine delivery systems, gene expression, inflammation, oral mucosa, Papanicolaou test

## Abstract

**Background:**

The packaging and marketing of electronic cigarettes (e‐cigs) often target younger demographics. This study aimed to evaluate gene expression in e‐cig users through exfoliative cytology.

**Methods:**

Samples were collected from 17 e‐cig users and 10 nonsmokers as controls. Clinical data included age, gender, heart rate, oximetry, capillary blood glucose, carbon monoxide levels, sialometry, alcohol‐related risk scores, alcohol consumption, and e‐cig use parameters. Smears from the left tongue edge were obtained using a Rovers Orcellex Brush. The Papanicolaou method assessed epithelial maturation and cytological features, categorized from normal to conclusive for malignancy. Cellular composition, inflammation, microbial presence, and atypia were evaluated using a semiquantitative scoring system. Gene expression (p16, IL1‐beta, CXCL8, TNF, and KRT13) was analyzed by RT‐PCR. Statistical comparisons used the Mann–Whitney test, and correlations were assessed via Spearman's test (*p* ≤ 0.05).

**Results:**

Fruit flavors were the most preferred. Some users were former smokers (average abstention: 3.15 months). Bacterial colonies were more prevalent in the e‐cig group (64.7% vs. 20%, *p* = 0.085), mucus and inflammatory changes were found exclusively in e‐cig users (*p* = 0.062). No significant differences were found in the Papanicolaou classification by gender (*p* = 0.904). Gene expression analysis showed a differential expression of p16 and TNF between the groups. Significant correlations were found between carbon monoxide and p16 expression (*r* = −0.41, *p* = 0.02), vaping sessions per day and p16 expression (*r* = −0.37, *p* = 0.02), and daily alcohol dose and TNF expression (*r* = −0.42, *p* = 0.04).

**Conclusion:**

E‐cigarette use may induce early molecular and cytological changes in the oral mucosa, affecting inflammation, immunity, and epithelial differentiation.

## 1 Introduction

The packaging and marketing strategies employed for electronic cigarettes (e‐cigs), also known as electronic nicotine delivery systems (ENDS) and heated tobacco products (HTPs), have attracted a lot of attention, becoming common among the young population [1]. This is mainly due to the strong commercial appeal, where the device comes in a variety of designs, colors, and flavorings, as well as producing large amounts of smoke, even without the direct burning of tobacco. This appeal to youth is a significant concern, as it may lead to increased experimentation and eventual addiction among adolescents. Widespread prevalence, especially among young people, underscores the need for awareness campaigns to warn of the risks and understand the potential harm [2].

While marketing restrictions for HTPs vary globally, there is a widespread debate concerning their potential to mitigate the health risks associated with tobacco use [2]. In Spain, tobacco companies have introduced HTPs under various brands. This has sparked contentious discussions within the country due to the contrast between stringent prohibitions on tobacco product marketing, including HTPs, and the absence of similar restrictions on e‐cigarettes, vaping products, or tobacco heating devices [3].

A study conducted by Schwarzmeier et al. has shown that e‐cig and alcohol users exhibit genotoxicity and cytotoxicity in oral mucosa cells [4]. Additionally, former smokers who use e‐cigs and alcohol may experience more damage to their oral mucosa cells compared to those who have not used e‐cigs.

The oral epithelium of e‐cig users showed deregulation of crucial genes and related molecular pathways, displaying both similarities and differences when compared to smokers. Tommasi et al. concluded that these findings have significant implications for public health and tobacco regulation [5].

Exfoliative cytology is a diagnostic and preventive tool for infectious, vesiculobullous, oral potentially malignant disorders, and malignant lesions of the oral cavity. In addition to being a noninvasive technique with advantages such as low cost and rapid execution, it is also more readily accepted by patients due to its painless nature [6]. This technique is associated with cytochemical processes that allow the morphological interpretation of the naturally or artificially desquamated epithelial cells from the oral mucosa or other mucosa. It has also been used very successfully in the molecular biology field, to obtain RNA and verify the expression of genes involved in the carcinogenesis process in smokers [7].

This study aims to explore the relationship between e‐cig use, clinical parameters, cytological alterations, and *p16*, *IL1‐beta*, *CXCL8*, *TNF*, and *KRT13* gene expression in exfoliated oral mucosal cells. The genes p16, IL1‐beta, CXCL8, TNF, and KRT13 were selected based on their known roles in inflammation, cell cycle regulation, and epithelial differentiation.

## 2 Materials and Methods

### 2.1 Participants and Sample Size Estimation

Patients were recruited from the oral medicine, oral surgery, and implantology unit through promotional campaigns on social media and the center's own channels. Patients who initially met the inclusion criteria attended the unit to be validated by the researchers. Control group participants were selected from among the center's own staff and other external sources and matched for age and sex.

Regarding the study's power estimate, the selection of p16 as the reference marker for the sample size estimation was based on its well‐established role in the literature as one of the most consistently altered genes in oral mucosal pathologies, particularly in relation to cellular dysregulation and exposure‐related molecular changes. Therefore, p16 was considered a robust and representative biomarker for estimating potential differences in gene expression between groups [8, 9, 10, 11]. Assuming a difference in means in the expression of the p16 gene between cases and controls of 58.16 with an SD of 45.16—values obtained from a pilot study conducted prior to the formal start of the project involving four samples from e‐cigarette users and four from nonusers (representing approximately 23% of the final sample size)—and a confidence level of 95% for a sample size of 27 subjects, the power achieved was 88.5%. If 30 patients had been included, the power would have been 92.7%. These pilot samples were employed to optimize and validate the RT‐qPCR methodology for all target genes, and their p16 expression data were used to inform the sample size calculation. This estimate was calculated using the Epidat 4.2 programme (SERGAS, Galicia, Spain).

Unstimulated saliva samples and exfoliative cytology were collected from 27 individuals with clinically healthy mucosa. The individuals were divided into two groups:

– E‐cigarette group: consisting of 17 regularly and exclusively e‐cig users, for at least 6 months.
– Control Group: 10 nonsmokers or e‐cig users.

### 2.2 Clinical Data

All participants underwent extra and intraoral physical examinations. The collected data included information on age, gender, heart rate (bpm), oximetry, capillary blood glucose levels, carbon monoxide levels in expired air using a piCO+ Smokerlyzer (Bedfont Scientific, Kent, England) (in parts per million), sialometry (saliva flow rate in mL per minute), alcohol‐related risk scores, and daily alcohol consumption (mL).

To perform the collections, individuals were instructed to abstain from brushing their teeth or eating food for 2 h. Additionally, they were required to refrain from consuming alcohol for 12 h [12].

### 2.3 Alcohol and E‐Cig Consumption

The patients' alcohol profile was established using the AUDIT test (Alcohol Use Disorder Identification Test) via an online questionnaire (Google Forms). Additionally, a second questionnaire in the same format was administered to the e‐cig group, which included inquiries about e‐cig consumption including e‐cig consumption period (in years), number of sessions per day, duration of each session (in minutes), daily e‐liquid quantity consumed (in milliliters), nicotine quantity per day (in milligrams per milliliter), and flavorings, as well as simultaneous e‐cig and alcohol use, and any observed increase in e‐cig use with alcohol consumption.

### 2.4 Saliva and Tongue Smears Collection

First, unstimulated saliva was collected in a sterile conical tube for 5 min. The saliva flow rate was then determined by measuring the saliva's volume (mL) per minute.

The smears collected from the left lateral border of the tongue were collected using a Rovers Orcellex Brush (Rovers Medical Devices, Oss, the Netherlands) by scraping the site vigorously rotating the brush 20 times and placed in sterile tubes containing 2 mL of Roswell Park Memorial Institute (RPMI) (Microvet, Madrid, Spain). The tubes were then placed on dry ice until stored in a −80°C freezer.

### 2.5 Papanicolaou Stain

**FIGURE 1:**
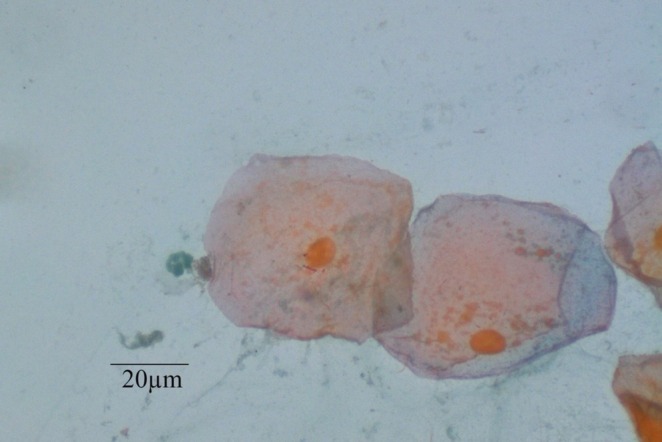
Photomicrograph showing two nucleated superficial epithelial cells containing keratohyalin granules, with a neutrophil on the left side.

**FIGURE 2:**
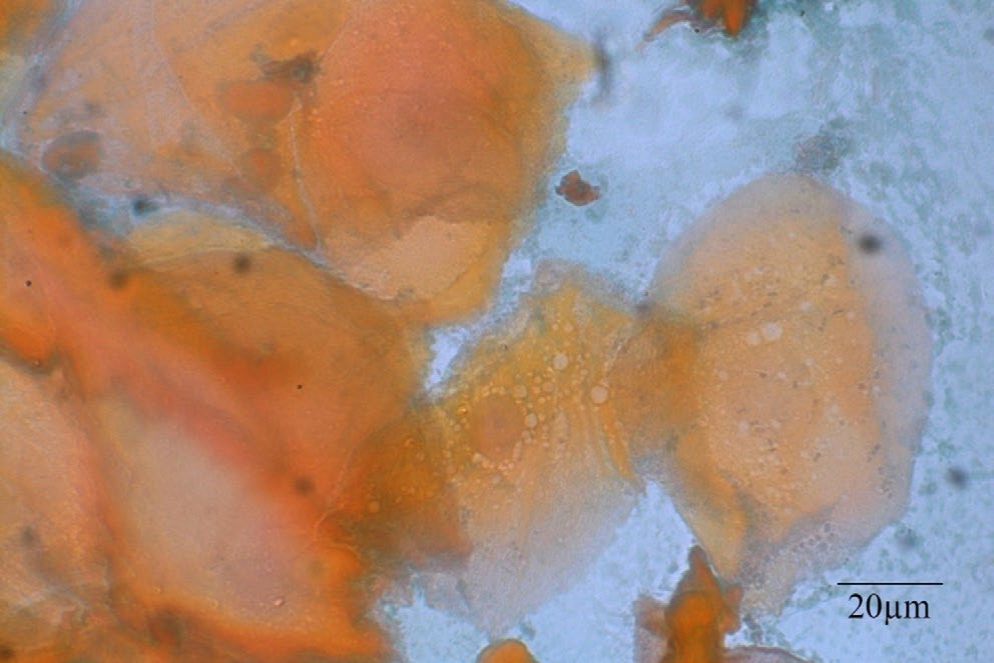
Cluster of superficial epithelial cells, some of which lack a nucleus. The cells on the right display cytoplasmic vacuolization.

The Papanicolaou method allows morphologic feature evaluation and the assessment of the pattern of epithelial cellular maturation. For qualitative criteria, the cytologic findings are rated as class 0 (not representative), class I (normal, absence of abnormal, or atypical cells), class II (normal with inflammatory alterations), class III (atypical, suggestive but inconclusive for malignancy), class IV (strongly suggestive, but not conclusive for malignancy), and class V (cytology conclusive for malignancy) [13]. These were stratified by the Papanicolaou classification, observing the presence of superficial cells with a nucleus; superficial cells without a nucleus; intermediate cells; basal cells; inflammatory infiltrate; inflammatory alterations (perinuclear halo, vacuolization, and color change); red blood cells, mucus, bacterial colonies, Candida hyphae, keratohyalin granules, and cellular atypia (Figures 1 and 2). The following occurrence scores were used: 0 = absent; 1 = up to 25%; 2 = 25%–50%; 3 = more than 50% [14].

### 2.6 Real‐Time Polymerase Chain Reaction (RT‐qPCR)

Total RNA was extracted using a Trizol kit (Ambion Inc., Carlsbad, CA, USA). After assessing purity and integrity with a spectrophotometer and agarose gel, respectively, 1000 ng of RNA was treated with DNase I Amplification Grade (Ambion Inc., Carlsbad, CA, USA) and transcribed into complementary DNA using the SuperScript III First‐Strand Synthesis System kit (Invitrogen, Carlsbad, CA, USA). qRT‐PCR reactions were performed under optimized conditions for each target. Genes exhibiting amplification beyond 34 PCR cycles were considered to have potential nonspecific amplification. This approach follows established qRT‐PCR guidelines, ensuring specificity and reliability of the results [15].

The sequences of primers TP16, IL1‐beta, CXCL8, TNF, and KRT13 were confirmed on the NCBI/Gene Bank website, ensuring specificity for *Homo sapiens* . Two reference genes (GAPDH and TUBA6) were tested, and TUBA6 was selected due to its lower variation between samples. The details of the primers used in this study are presented in Table 1.

**TABLE 1:** Description of the primers with the following sense and antisense primers and their melting temperatures, the size of the amplified base pairs, and references.

Gene	Primers	Melting temperature (°C)	Base pairs	Amplicon size (bp)	FASTA PUBMED
*TUBA6*	F‐ CCGGGCAGTGTTTGTAGACT	60	20	99	NC_000012.12
	R‐ TTGCCTGTGATGAGTTGCTC	58	20		
*TP16*	F‐ GATCCAGGTGGGTAGAAGGTC	54	21	74	NC_000009.12
	R‐ CCCCTGCAAACTTCGTCCT	55	19		
*IL1B*	F‐ ATGATGGCTTATTACAGTGGCAA	53	23	132	NC_000002.12
	R‐ GTCGGAGATTCGTAGCTGGA	54	20		
*CXCL8*	F‐ TTTTGCCAAGGAGTGCTAAAGA	53	22	194	NC_000004.12
	R‐ AACCCTCTGCACCCAGTTTTC	56	21		
*TNF*	F‐ CCTCTCTCTAATCAGCCCTCTG	54	22	220	NC_000006.12
	R‐ GAGGACCTGGGAGTAGATGAG	54	21		
*KRT13*	F‐ GACCGCCACCATTGAAAACAA	55	21	177	NC_000017.11
	R‐ TCCAGGTCAGTCTTAGACAGAG	53	22		

The SYBR Green fluorophore (Platinum SYBR Green qPCR SuperMix‐UDG, Applied Biosystems, Framingham, MA, USA) was utilized with the StepOnePlus System (Applied Biosystems, Framingham, MA, USA). The cDNA product in the exponential phase of the amplification reaction was quantified using the following cycling parameters: 50°C for 2 min, an initial denaturation at 95°C for 2 min, followed by 40 cycles of 95°C for 15 s and 60°C for 30 s. Relative gene expression was determined using the 2^−ΔΔC*t*^ method [7].

### 2.7 Statistical Analysis

Data were analyzed using SPSS version 23 (SPSS Inc., Chicago, IL, USA). Papanicolaou analysis by groups was conducted using Chi‐Square and ANOVA tests. Normality for the genes was assessed using the Shapiro–Wilk test.

The statistical significance of gene expression between groups was assessed using the Mann–Whitney test (GraphPad Software 6.00, San Diego, CA, USA). Correlation analysis between gene expression and clinical data was performed using Spearman's test. A *p* ≤ 0.05 was considered to indicate statistical significance.

## 3 Results

### 3.1 Sample Characteristics

Samples were obtained from a total of 27 individuals, consisting of 17 e‐cig users and 10 nonsmokers (control group). There were 15 women and 12 men, with a mean age of 39 years. The gender distribution (female and male) and age between the groups was not statistically significant (Table 2).

**TABLE 2:** Demographic and clinical characteristics by group. *p* value: ANOVA.

	E‐Cig		Control		*p*
	Mean	SD	Mean	SD	
Men	8	47.1	4	40.0	0.822
Women	9	52.9	6	60.0	
Age	38.18	12.14	40.20	15.25	0.707
Heart rate	74.59	11.49	73.40	16.85	0.829
Oximetry	93.71	7.20	98.00	0.82	0.074
Capillary blood glucose	93.24	21.27	88.40	12.47	0.520
Carbon monoxide (ppm)	1.44	0.89	3.40	2.46	0.006
Sialometry (mL/min)	4.02	0.81	3.95	1.61	0.885

A higher average heart rate was observed in the e‐cig group (74.59 vs. 73.40), although it was not statistically significant. The oximetry readings were an average of 98 in the control group compared to 93.71 in the e‐cig group (*p* = 0.074). Additionally, the average blood glucose level was higher in the vaping group compared to the control group (93.24 vs. 88.4). No significant differences between groups were observed regarding sialometry (Table 2).

#### 3.1.1 E‐Cig Consumption

Table 3 displays data related to e‐cigarette consumption. Among e‐cig consumers, flavor preferences varied, with six individuals favoring fruit flavors, two preferring caramel, one each opting for vanilla, biscuit, mint, and ice. Additionally, five participants did not specify a preference. Notably, some e‐cig users were former traditional cigarette smokers, with an average abstention period of 3.15 months (SD = 2.19).

**TABLE 3:** Descriptive data on e‐cig group consumption.

		E‐cig *n* = 17
E‐cig consumption period (years)	Mean	3.29
	Standard deviation	2.85
Number of sessions per day	Mean	2.24
	Standard deviation	1.20
Time for each session (minutes)	Mean	1.43
	Standard deviation	1.02
E‐liquid quantity/day (mL)	Mean	6.75
	Standard deviation	9.63
Nicotine quantity/day (mg/mL)	Mean	5.89
	Standard deviation	5.42
Simultaneous use of e‐cig and alcohol	No	2
	Yes	14
	Sometimes	1
Increase in e‐cig use with alcohol consumption	No	8
	Yes	9

#### 3.1.2 Exfoliative Cytology

Table 4 provides an overview of the exfoliative cytology variables analyzed by group. Although none of the differences reached statistical significance, several findings were close to significance. Bacterial colonies were detected in 64.7% of the e‐cig group compared to 20% of the control group (*p* = 0.085). Mucus was present in 25%–50% of the smear exclusively in the e‐cig group (*p* = 0.062). Additionally, all cases with inflammatory changes belonged to the vaping group (*p* = 0.062). No significant differences were observed in the Papanicolaou classification when analyzed by gender (*p* = 0.904).

**TABLE 4:** Exfoliative cytology variables by group *p* value: Chi square.

		E‐cig (*n* = 17)		Control (*n* = 10)		*p*
		*N*	%	*N*	%	
Presence of superficial cells with a nucleus	Up to 25%	4	23.5	0	0	0.169
	25%–50%	1	5.9	2	20.0	
	> 50%	12	70.6	8	80.0	
Superficial cells without a nucleus	Up to 25%	12	70.6	6	60.0	0.813
	25%–50%	3	17.6	2	20.0	
	> 50%	2	11.8	2	20.0	
Intermediate cells	Absent	7	41.2	5	50.0	0.523
	Up to 25%	8	47.1	5	50.0	
	25%–50%	2	11.8	0	0	
Basal cells	Absent	17	100	10	100	
Inflammatory infiltrate	Absent	6	35.3	1	10.0	0.222
	Up to 25%	10	58.8	9	90.0	
	25%–50%	1	5.9	0	0	
Perinuclear halo	Absent	14	82.4	9	90.0	0.589
	Up to 25%	3	17.6	1	10.0	
Vacuolization	Absent	7	41.2	5	50.0	0.656
	Up to 25%	10	58.8	5	50.0	
Color change	Absent	3	17.6	9	90.0	0.743
	Up to 25%	13	76.5	1	10.0	
	25%–50%	1	5.9	0	0	
Red blood cells	Absent	17	100	9	90.0	0.184
	Up to 25%	0	0	1	10.0	
Mucus	Absent	4	23.5	0	0	0.062
	Up to 25%	10	58.8	10	100	
	25%–50%	3	17.6	0	0	
Bacterial colonies	Absent	6	35.3	8	80.0	0.085
	Up to 25%	8	47.1	1	10.0	
	25%–50%	3	17.6	1	10.0	
Candida hyphae	Absent	14	82.4	7	70.0	0.456
	Up to 25%	3	17.6	3	30.0	
Keratohyalin granules	Absent	5	29.4	6	60.0	0.118
	Up to 25%	12	70.6	4	40.0	
Cellular atypia	Absent	17	100	10	100	
Papanicolaou classification	Class 0	4	23.5	0	0	0.062
	Class I	10	58.8	10	100	
	Class II	3	17.6	0	0	

### 3.2 Gene Expression

**FIGURE 3:**
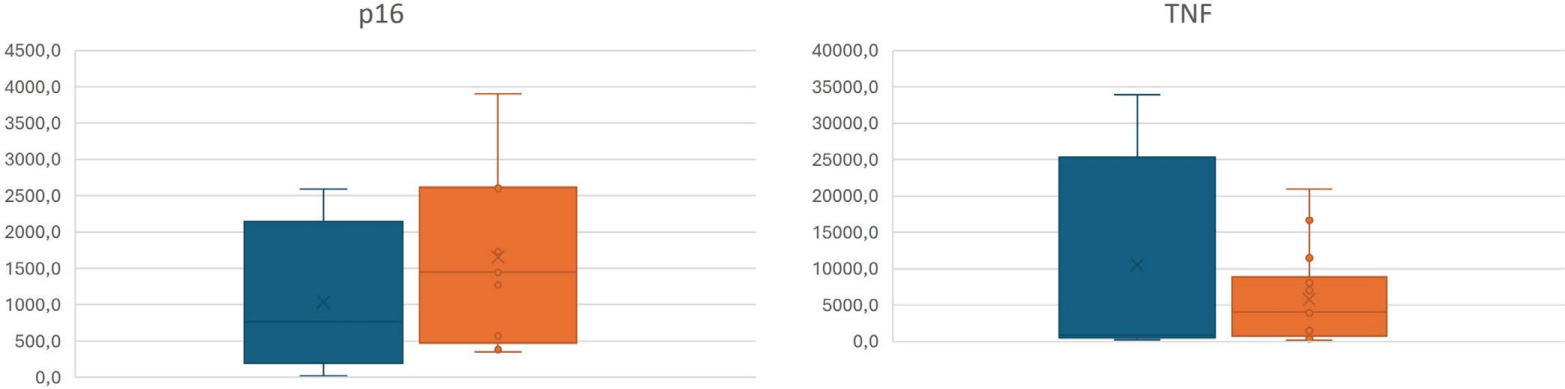
Boxplots of p16 and TNF expression across cohorts. Blue: controls, orange: e‐cig group.

Genes exhibiting amplification beyond 34 PCR cycles were considered to have nonspecific amplification; therefore, these genes were excluded from the analysis. For the five reliably amplified transcripts, reaction conditions were optimized and amplification efficiencies were confirmed to be comparable to the reference gene, validating the use of the 2^−ΔΔC*t*^ method for relative quantification. The control group showed lower p16 expression compared to the e‐cigarette group (*p* = 0.45). Although TNF expression was higher in the control group (*p* = 0.92) than in the vaping group, neither difference was statistically significant (Figure 3).

Genes were found to be negatively regulated in the e‐cig group compared to the control group. Significantly, correlations were observed between certain variables: carbon monoxide (ppm) and *p16* gene expression (Spearman's correlation coefficient = −0.41; *p* = 0.02), number of sessions per day and *p16* gene expression (Spearman's correlation coefficient = −0.37; *p* = 0.02), and alcohol dose per day and expression of the *TNF* gene (Spearman's correlation coefficient = −0.42; *p* = 0.04).

The relationship between cytopathological changes observed in the Papanicolaou classification, p16 and TNF gene expression, demonstrated no statistical significance between the e‐cig and control groups (*p* = 0.608 for p16; and *p* = 0.164 for TNF).

## 4 Discussion

The indiscriminate use of e‐cig, without adequate potential health risk consideration or knowledge of their association with oral cancer development, poses a significant public health concern. While research on the effects of e‐cigs in pulmonary diseases has advanced over the past decade, studies focusing on their impact on the oral mucosa remain limited. Moreover, there is no clear consensus on how these devices affect oral tissues.

Our patients smoked exclusively e‐cigs for an average of 3.29 years. Sobieski et al. observed that after 2.4 years, most long‐term users (88.7%) continued exclusive e‐cig use, with only 7.7% quitting [16]. These findings suggest that e‐cigarette use patterns can persist for years, reducing the likelihood of them being used as an aid in smoking cessation efforts. Etter and Bullen concluded that e‐cig use may contribute to relapsing prevention in former smokers and smoking cessation in current smokers, however most of those patients who were daily e‐cig users continued vaping after 1 year [17]. Regarding flavor preferences, our data aligns with the systematic review by Zare [18], which reported that adults generally predominantly prefer sweet flavors. Similarly, our results are consistent with those of Carvalho et al. [19]. When analyzing flavor preference changes in a cohort of long‐term e‐cig users, a shift toward sweet flavors was observed, particularly among younger individuals and exclusive e‐cig users [20].

E‐cig users reported an average of 2.24 sessions per day, with a mean duration of 1.43 min per session. Some studies highlight substantial variability in e‐cig consumption patterns, emphasizing the need to consider individual characteristics and behaviors when assessing the health effects of e‐cig use [21]. Kosmider et al. reported that daily e‐cig users typically take 156–163 puffs per day, often grouped into 10–12 puffs per session [21].

Nicotine delivery efficacy varies across brands and models of ENDS [22]. However, the nicotine levels delivered are comparable to or even higher than those of traditional cigarettes, with similar systemic retention [23]. In the present study, the average daily nicotine consumption was 5.89 mg/mL. Behar et al. reported nicotine intake ranging from 1.2 to 1.4 mg per session, values comparable to conventional cigarettes but lower than those observed in our findings [24].

Additionally, the nicotine amount vaporized depends on the type of cartridge used [22], and the nicotine concentration in e‐liquids significantly influences delivery efficiency [24]. Participants in our study reported an average e‐liquid daily consumption of 6.75 mL. Given that the mean nicotine concentration in e‐liquids is approximately 11 mg/mL, this corresponds to an estimated daily exposure of 0.38 mg/kg of body weight [25]. Nicotine delivery efficiency also varies considerably among e‐cig brands, with total nicotine levels in vapor ranging from 0.5 to 15.4 mg per 20 series of 15 puffs [22]. On average, 50%–60% of the nicotine in a cartridge is vaporized [22].

Xerostomia is the most frequently reported oral complaint associated with ENDS use [26]. Two recent systematic reviews published in 2023 [27, 28], concluded that e‐cig consumption may increase susceptibility to dental caries, periodontal disease, and mucosal alterations, as well as contribute to tooth and prosthesis discoloration. Sialometry in the present study did not reveal significant differences between the e‐cig and control groups. Since xerostomia is a subjective symptom, its presence may not necessarily correlate with saliva flow rates.

Heated nicotine aerosols from ENDS contain various tobacco‐specific nitrosamines, which are considered potential carcinogens and can induce harmful alterations in oral cavity cells. A 2024 literature review reaffirmed previous findings, indicating an increased risk of periodontitis and caries associated with vape use [2].

Although in vitro studies provide insights into the potential mechanisms underlying cellular alterations induced by e‐cig use, observational clinical studies of the oral mucosa—before the onset of clinically visible lesions—can offer a better understanding of cellular pathways activation related to inflammation, keratinization, senescence, DNA repair, and proliferation. Additionally, salivary metabolite alterations have been identified in e‐cig users, particularly with elevated levels of metabolites associated with inflammation, xenobiotic metabolism, and biomass‐burning pathways [19].

In our study, all analyzed smears from the vaping group exhibited inflammatory alterations. This aligns with findings from Pavanello et al., who reported a higher proportion of inflammatory cells in cigarette smokers [29], and Sepulveda Inostroza et al., who observed similar changes in hookah users [14]. However, previous studies, such as that of Schwarzmeier et al., were limited by the inclusion of patients with alcohol and cigarette smoking histories, making it difficult to attribute findings solely to e‐cig use [4]. In the present study, among the 17 participants in the e‐cig group, 14 reported concurrent alcohol consumption, and nine indicated that alcohol intake increased their e‐cig use.

Chhina et al. reported that although e‐cig contain fewer carcinogens than traditional cigarettes, concerns remain regarding their potential for DNA damage [30]. While a direct causal link between e‐cig use and oral cancer has not been established, case report of oral cancer in a heavy e‐cig user without other traditional risk factors has been documented.

In our study, no statistically significant differences were observed in p16 or TNF expression between groups [7]. Although the mean p16 was higher in the e‐cig group (*p* = 0.45), this increase might reflect an adaptive response to epithelial stress (senescence or cell cycle arrest) rather than a pro‐carcinogenic event. In classic oral carcinogenesis, CDKN2A inactivation and consequent p16 downregulation are more frequently described, whereas p16 overexpression is more characteristic of contexts involving RB pathway disruption (e.g., HPV‐associated tumors) and inflammatory processes [31]. The absence of differences in TNF (*p* = 0.92) suggests that, at least with these markers, no clear inflammatory or pro‐neoplastic pattern attributable to vaping is detected. These results should be interpreted with caution due to the sample size and observed variability. There is substantial evidence that chronic inflammation caused by chemical, bacterial, or viral agents increases carcinogenic risk. This occurs through the accumulation of cytokines, chemokines, prostaglandins, reactive oxygen species (ROS), and nitrogen radicals in chronically inflamed tissues, which can induce cell proliferation and promote prolonged cell survival by activating oncogenes and inactivating tumor suppressor genes. The relationship between inflammation and tobacco smoke exposure has been demonstrated both in oral submucous fibrosis and in in vitro studies of lung cell lines exposed to e‐cig vapor. Contact with areca and betel nut or e‐cig vapor has been shown to stimulate the production of growth factors, prostaglandins, ROS, TNF‐α, IL‐8, IL‐6, TGF‐β, and other cytokines and chemokines, thereby increasing the risk of malignant transformation in oral mucosal cells [31]. Additionally, these exposures inhibit phagocytosis, making tissues more susceptible to infection, and suppress cellular antioxidant defenses, leading to significant DNA damage [32].

Our results are consistent with a previous study by Tsai et al. which reported differential regulation of oral squamous cell carcinoma (OSCC) cell invasion and inflammatory effects following exposure to e‐cig flavorings and nicotine [33]. Similarly, a pilot study by Hamad et al. found that vaping altered *TP53* expression using RT‐PCR [34].

Furthermore, we observed negative correlations between exhaled carbon monoxide levels and *p16* gene expression, as well as between the frequency of daily e‐cig sessions and *p16* expression. This suggests that increased exposure to e‐cig constituents, such as carbon monoxide, may suppress genes involved in cell cycle regulation and DNA repair, potentially promoting carcinogenesis [4].

Additionally, a negative correlation was found between daily alcohol consumption and *TNF* gene expression, suggesting a synergistic interaction between e‐cig use and alcohol intake in exacerbating oral mucosal damage. This raises even greater concern, as simultaneous use of e‐cig and alcohol is highly prevalent among college students, with over 89% reporting either simultaneous or nonsimultaneous use [35]. These findings underscore the importance of considering lifestyle factors when assessing health risks associated with e‐cig use.

While our results are consistent with previous studies describing cellular and genotoxic impairments in e‐cig and alcohol users, this investigation uniquely contributes by identifying specific gene regulatory patterns in the oral mucosa and their correlation with consumption behaviors. Although further research is needed to conclusively determine the oncogenic potential of these alterations, our findings suggest that e‐cig may pose greater health risks than commonly assumed, emphasizing the need for caution in their use.

Despite these insights, our study has limitations, including a relatively small sample size and its cross‐sectional design, which precludes causal inferences. Due to the sample size selected, it has not been possible to perform a stratified analysis by age, which may introduce some bias in the age‐related alterations of the oral mucosa. Additionally, cytopathological evaluation can be subjective, potentially leading to variations in classification interpretations, which may affect the reproducibility of results. Future research should focus on assessing the long‐term effects of e‐cigarette use on oral and systemic health.

## 5 Conclusion

In conclusion, the present study provides novel insights into the cytopathological and molecular impacts of ENDS on the oral mucosa. By combining cytological analysis with gene expression profiling, we demonstrate that e‐cig use is associated with inflammatory alterations and a significant downregulation of key genes involved in immune response, inflammation, and epithelial integrity. Despite the confounding factor of alcohol consumption in part of the cohort, the consistency of cytological alterations and the molecular suppression of markers such as p16 and TNF point to a potentially deleterious effect of e‐cigarettes on oral health. These findings underscore the need for further investigation into the long‐term oral effects of e‐cigarettes, particularly in younger populations who may perceive these devices as a safer alternative to conventional tobacco.

## Author Contributions

**Alba Pérez‐Jardón:** data curation, formal analysis, investigation, methodology, software, visualization, writing – original draft. **Bruna Fernandes do Carmo Carvalho:** conceptualization, data curation, methodology, software, visualization, funding acquisition. **Cintia Micaela Chamorro‐Petronacci:** investigation, methodology. **Maria Dolores Reboiras‐López:** investigation, methodology. **Natalia de Carvalho Faria:** data curation, methodology, writing – review and editing. **Renata Falchete do Prado:** data curation, formal analysis, methodology, validation, writing – original draft, writing – review and editing. **Mónica Ghislaine Oliveira Alves:** conceptualization, data curation, formal analysis, methodology, supervision, validation, writing – original draft, writing – review and editing. **Fábio França Vieira e Silva:** investigation, methodology. **Maria‐Elena Padín‐Iruegas:** investigation, methodology. **Mario Pérez‐Sayáns:** conceptualization, data curation, formal analysis, investigation, resources, supervision, validation, writing – original draft, writing – review and editing, project administration. **Janete Dias Almeida:** conceptualization, investigation, resources, supervision, writing – original draft, writing – review and editing, project administration, funding acquisition. All authors have read and agreed to the published version of the manuscript.

## Funding

This work was supported by the Fundação de Amparo à Pesquisa do Estado de São Paulo (#2020/10362‐0, #2020/10322‐9) and the Coordenação de Aperfeiçoamento de Pessoal de Nível Superior (001).

## Ethics Statement

This study was approved by the Ethics Committee for Research Involving Human Subjects at ICT‐UNESP (protocol number: 4.397.780). The principles outlined in the Declaration of Helsinki on clinical research involving human subjects were followed. Written informed consent was obtained from each enrolled patient.

## Consent

Informed consent was obtained from all subjects involved in the study.

## Conflicts of Interest

The authors declare no conflicts of interest.

## Data Availability

The data that support the findings of this study are available on request from the corresponding author. The data are not publicly available due to privacy or ethical restrictions.
